# Distinct Structural Responses of Lipid Bilayers to Horizontal and Vertical Electric Fields

**DOI:** 10.1021/acs.jpclett.5c02764

**Published:** 2025-10-06

**Authors:** Hironori Kageyama, Maki Komiya, Eiji Yamamoto, Ayumi Hirano-Iwata

**Affiliations:** † Graduate School of Biomedical Engineering, 13101 Tohoku University, 6-6-12, Aramaki Aza Aoba, Aoba-ku, Sendai, Miyagi 980-8579, Japan; ‡ Research Institute of Electrical Communication (RIEC), 13101 Tohoku University, 2-1-1, Katahira, Aoba-ku, Sendai, Miyagi 980-8577, Japan; § Department of System Design Engineering, 12869 Keio University, 3-14-1 Hiyoshi, Kohoku-ku, Yokohama, Kanagawa 223-8522, Japan; ∥ Advanced Institute for Materials Research (WPI-AIMR), 13101 Tohoku University, 2-1-1 Katahira, Aoba-ku, Sendai, Miyagi 980-8577 Japan

## Abstract

Electric fields modulate the structure and mechanical properties of biological membranes, influencing diverse cellular processes. While the effects of vertical electric fields (***E***_Vert_) across the membranes have been extensively studied, the influence of horizontal (in-plane) electric fields (***E***_Horz_) remains poorly understood. Here, using molecular dynamics (MD) simulations and bright-field imaging of planar lipid bilayers (PLBs), fundamental components of biological membranes, we investigate how ***E***_Horz_ and ***E***_Vert_ affect lipid bilayer properties. Simulations revealed that ***E***_Horz_ induces greater structural changes than ***E***_Vert_, such as membrane area reduction and increases in lipid tail ordering, even at high cholesterol concentrations. Consistently, bright-field imaging shows that the DC horizontal membrane voltage (*V*_Horz_) causes membrane area contraction, whereas the vertical voltage (*V*_Vert_) has no effect under the tested conditions. These results demonstrate that in-plane electric fields elicit distinct structural responses in lipid bilayers, highlighting its potential physiological relevance and importance in membrane biophysics.

## Graphical abstract

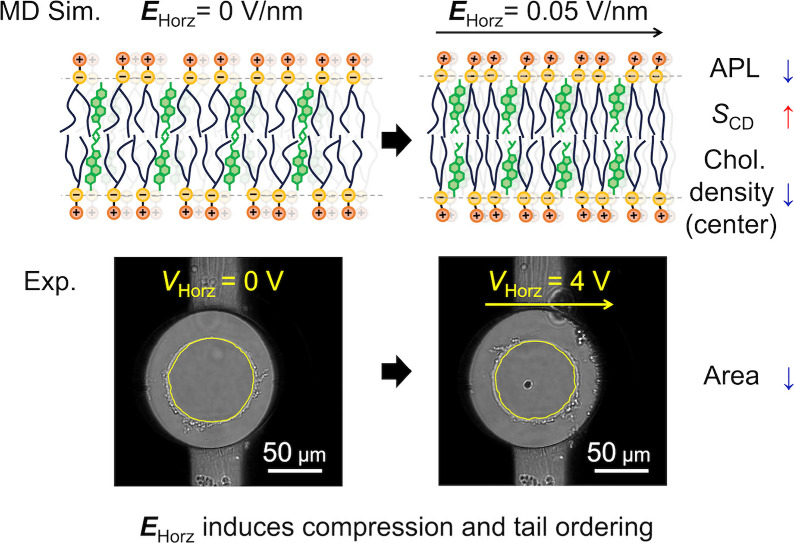

The lipid bilayer, a fundamental component of biological membranes, plays a central role in maintaining membrane integrity, regulating transport processes, and modulating signal transduction. Its structural and mechanical properties, such as thickness, curvature, tension, and bending rigidity, directly influence the conformational dynamics and activity of embedded proteins by shaping their local lipid environment.^1–6^ Lipid membranes are now widely recognized as active regulators of cellular function rather than as passive matrices.^7–10^ Electric fields modulate the membrane behavior in a wide range of biological contexts. In addition to their classical role in transmembrane signaling, electric fields applied perpendicular to the membrane (***E***_Vert_) have been implicated in development,^11–13^ regeneration,^14–16^ and cancer proliferation.^17–20^ These electric fields arise from electrochemical gradients of membrane-permeable ions, which generate a potential difference across the membrane.^21,22^ In addition to ***E***_Vert_, horizontal (in-plane) electric fields (***E***_Horz_) also arise under physiological conditions. ***E***_Horz_ is not limited to specialized experimental conditions or pathological states but rather a ubiquitous feature of biological systems.

There are two main physiological scenarios in which ***E***_Horz_ can arise. First, static ***E***_Horz_ exists in epithelial cells, which constitute the predominant cell population in human tissues.^23^ Epithelial cells maintain transepithelial potential differences ranging from 5 to 50 mV across tight junctions, implying that the plasma membranes at tight junctions are indeed exposed to ***E***_Horz_,^24^ a phenomenon generally observed in ion-transporting epithelia.^25,26^ Second, dynamic ***E***_Horz_ can arise transiently during rapid ion movement through confined regions such as ion channels or nanodomains. In neurons, Martí et al. reported that ion flux through ion channel pores generates intramembrane ***E***_Horz_ exceeding 10^–^ ^2^ V/nm.^27,28^ Groves et al.^29^ further modeled how a single moving charge can produce similar fields capable of reorganizing lipids over nanometer scales within microseconds. These electrostatic effects differ fundamentally from mechanical stretching and may uniquely modulate the membrane structure and protein function. Such a modulation of membrane properties could, in turn, influence the activity of membrane proteins. Our previous work demonstrated that ***E***_Horz_ can enhance the activity of the voltage-gated sodium channels Na_V_1.5 in planar lipid bilayers (PLBs), suggesting that ***E***_Horz_ may modulate protein function via alterations in bilayer structure.^30,31^ However, the molecular mechanisms underlying this effect remain unclear. In this study, we combine molecular dynamics (MD) simulations and bright-field imaging of PLBs to investigate how ***E***_Horz_ modulates the lipid bilayer structure, providing insights into its possible physiological relevance.

The MD simulations enabled direct quantification of molecular-scale parameters, including lipid packing, tail ordering, and area per lipid (APL) under varying electric field orientations and cholesterol concentrations. Complementarily, bright-field microscopy provided label-free, noninvasive, and real-time measurements of the membrane area. Together, this dual approach allowed us to examine the electromechanical effects of ***E***_Horz_ from both theoretical and experimental perspectives. The simulated systems consisted of lipid bilayers composed of 1,2-dioleoyl-*sn*-glycero-3-phosphocholine (DOPC) and cholesterol (Figures S1), solvated with TIP3P water and neutralized with 150 mM KCl (see Figure 1a). The cholesterol content was set to 0, 10, 20, or 30 mol %, defined as the molar ratio *N*_Chol_/(*N*_DOPC_ + *N*_Chol_) (Table S1), thus encompassing the fluid phase and approaching conditions associated with increased cholesterol-induced ordering, although not reaching the cholesterol-condensed phase typically observed in saturated lipid systems. To elucidate how externally applied electric fields modulate the lipid bilayer structure and mechanics, we conducted all-atom MD simulations under horizontal (***E***_Horz_) and vertical (***E***_Vert_) electric field conditions. The field strength in the simulations was 0.05 V/nm, which is well below the typical electroporation threshold of ∼0.5–1.0 V/nm reported in previous studies.^32–34^ MD simulations were performed using the GROMACS^35^ (version 2022.6) with the CHARMM36 force field.^36,37^ Production runs of 700 ns were carried out for each system, and the final 600 ns of each trajectory were analyzed (see details in the Supporting Information).

**Figure 1:**
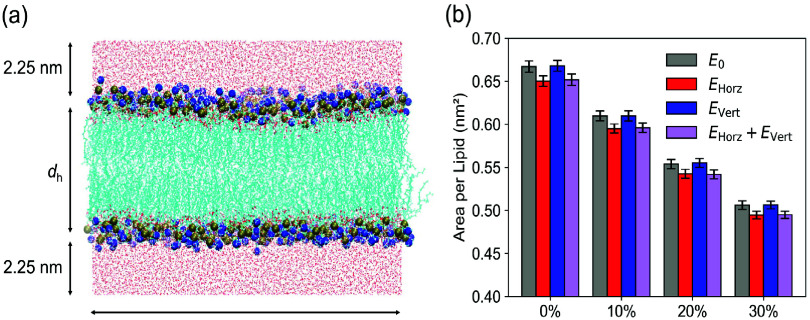
Simulation setup and structural effects of ***E***_Horz_ and ***E***_Vert_ on membrane area. (a) Side view of the DOPC–cholesterol bilayer system for molecular dynamics (MD) simulations. Carbon atoms are shown as light blue lines, phosphorus atoms as ocher spheres, nitrogen atoms as blue spheres, and water molecules as red dots. K^+^ and Cl^–^ ions are omitted for clarity. (b) Area per lipid (APL) at varying cholesterol concentrations (0, 10, 20, and 30 mol %) under different electric field conditions. Bars represent mean ± standard deviation over the final 600 ns of MD trajectories. Gray: no field; red: ***E***_Horz_ = 0.05 V/nm; blue: ***E***_Vert_ = 0.05 V/nm; purple: simultaneous ***E***_Horz_ and ***E***_Vert_ (each 0.05 V/nm).

The area per lipid (APL) was calculated as APL = *L*_*x*_ × *L*_*y*_/(*N*_DOPC_ + *N*_Chol_), where *L*_*x*_ and *L*_*y*_ are the box dimensions in the *x*- and *y*-directions, and *N*_DOPC_ and *N*_Chol_ are the number of DOPC and cholesterol molecules in a single leaflet, respectively. The equilibration of the simulated membranes was confirmed by monitoring the time variation of the APL, which stabilized after approximately 100 ns (Figure S2). In the absence of electric fields, the APL of the pure DOPC bilayer was 0.667 ± 0.007 nm^2^ (Figure 1b). Application of an ***E***_Horz_ = 0.05 V/nm reduced the APL to 0.650 ± 0.006 nm^2^, corresponding to a 2.6% decrease. In contrast, ***E***_Vert_ had no significant effect on the membrane area (APL: 0.668 ± 0.006 nm^2^). This reduction in APL under ***E***_Horz_ is consistent with a previous study,^38^ which reported a 2.8% decrease at the same field strength in pure DOPC membranes. Under combined ***E***_Horz_ and ***E***_Vert_ conditions, the APL was 0.652 ± 0.007 nm^2^, which is close to the value observed under ***E***_Horz_ alone, indicating that the horizontal field remains the dominant factor in modulating the membrane area. Although ***E***_Vert_ appears to partially mitigate membrane contraction induced by ***E***_Horz_, its influence is relatively minor. As shown in Figure 1b, similar trends were observed across cholesterol-containing membranes. In all cases, increasing cholesterol content decreased APL, consistent with the well-known ordering effect of cholesterol on lipid acyl chains.^39–42^ ***E***_Horz_ further reduced APL at each cholesterol level (up to 30 mol %), whereas ***E***_Vert_ again yielded negligible effects. These results indicate that ***E***_Horz_ induces in-plane compressive stress that reduces the membrane area, while ***E***_Vert_ does not produce significant mechanical alterations.

To assess the ordering of acyl chains, we calculated the deuterium order parameter (*S*_CD_) for DOPC tails using these equations:

$$-S_{\mathrm{CD}}=\frac{2}{3}S_{xx}+\frac{1}{3}S_{yy}$$

$$S_{ab}=\left\langle \frac{3\cos\;\theta_{a}\cos\;\theta_{b}-\delta_{ab}}{2}\right\rangle$$

where θ_a_ and θ_b_ are the angles between the molecular axis and the reference axes *a* and *b* (i.e., *x*, *y*, *z*) and δ_ab_ is the Kronecker delta.^43,44^ *S*_CD_ values range from 0 (disordered) to 0.5 (ordered), with lower values reflecting flexible tail segments and higher values indicating more rigid and aligned tails.^45,46^ As shown in Figure 2a,b, the *S*_CD_ profile for DOPC in the absence of cholesterol exhibits a gradual decline along the acyl chains, indicating increased flexibility toward the terminal methyl groups. A sharp minimum appears near carbon atoms 9–10, which corresponds to the cis-double bond in the acyl chains and is a disruption point in lipid ordering. These results are consistent with previously reported profiles from both simulation and experiment.^47,48^ Without electric fields, *S*_CD_ increased with cholesterol concentration, reflecting the ordering effect of cholesterol (see Figures S3 and S4). Upon application of ***E***_Horz_, the *S*_CD_ profile shifted upward across the entire acyl chain, indicating an enhanced tail alignment and reduced segmental flexibility (Figure 2c,d). This field-induced ordering was observed in both cholesterol-free and cholesterol-containing membranes. The increase was more pronounced in the terminal segments (C12–17), where *S*_CD_ values rose by more than 8–9%, suggesting that ***E***_Horz_ preferentially aligns the flexible tail ends along the membrane plane. In contrast, ***E***_Vert_ had a minimal effect, with profiles remaining nearly unchanged from the no-field condition. Furthermore, the *S*_CD_ profiles under combined ***E***_Horz_ and ***E***_Vert_ closely match those under ***E***_Horz_ alone, indicating that ***E***_Horz_ primarily governs the ordering effect. These results are consistent with the observations of Pogharian et al.,^38^ who also reported enhanced lipid ordering under lateral electric fields. Although lower *S*_CD_ values were observed in this study, despite using the same lipid type and CHARMM force field, this discrepancy is likely due to the temperature difference: 300 K in the present study and 260 K in their simulations. To validate our findings under physiologically relevant conditions, we compared the no-field (***E*** = 0 V/nm) profiles with zero-field studies by Akhunzada et al.^49^ (303 K) and Zoni et al.^50^ (298.15 K), both of which reported comparable values and profile shapes. These results support the reliability of our structural analysis. Although *S*_CD_ increased in response to ***E***_Horz_, the values remained within the range of the fluid phase, suggesting that no phase transition occurred. The structural effects of ***E***_Horz_ were consistent across varying cholesterol contents, highlighting distinct ways in which ***E***_Horz_ and ***E***_Vert_ modulate the membrane mechanics.

**Figure 2:**
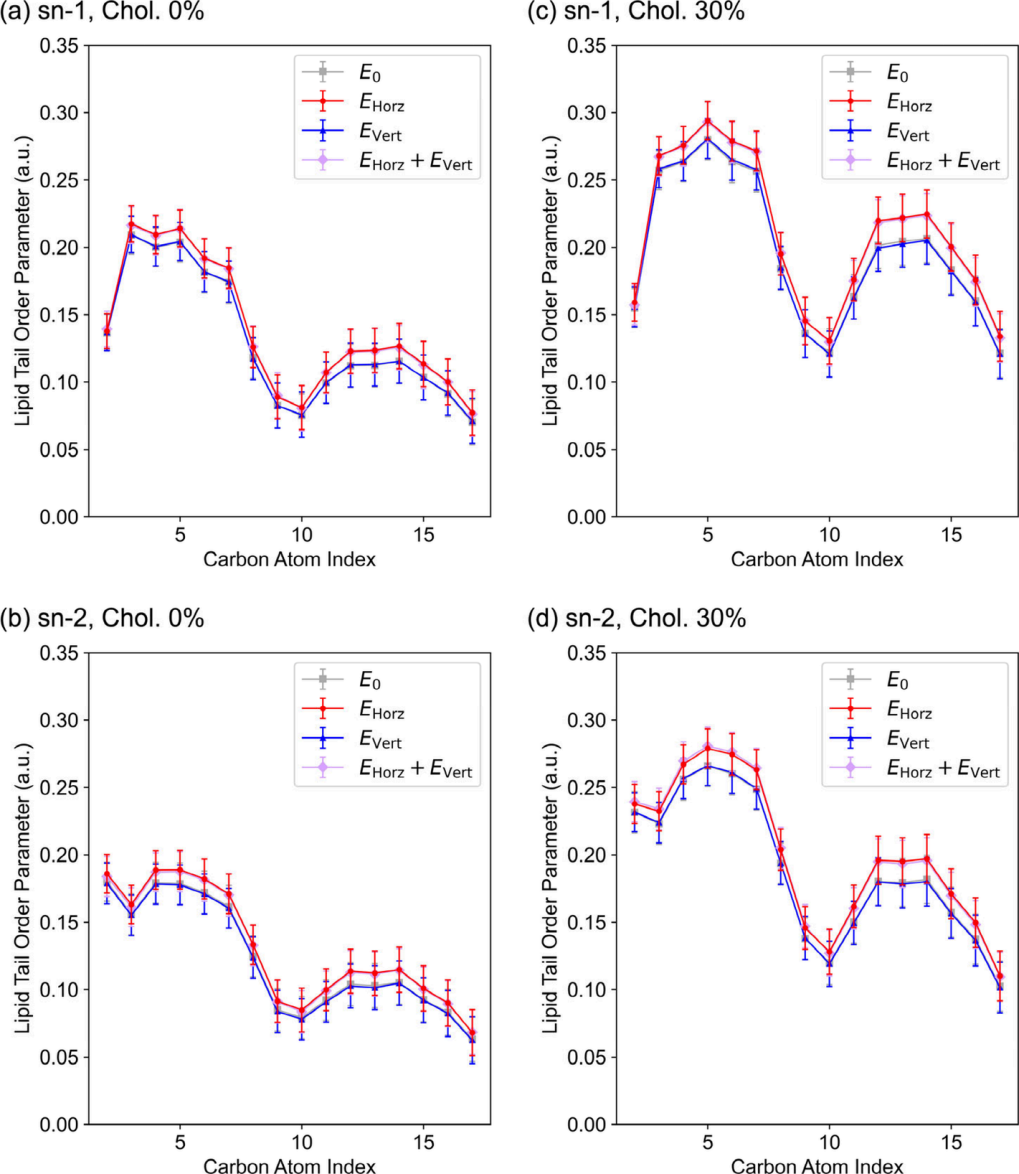
Effects of ***E***_Horz_ and ***E***_Vert_ on lipid tail ordering. Segmental order parameters (*S*_CD_) of DOPC hydrocarbon chains are plotted. Gray: no field; red: ***E***_Horz_ = 0.05 V/nm; blue: ***E***_Vert_ = 0.05 V/nm; purple: simultaneous ***E***_Horz_ and ***E***_Vert_ (each 0.05 V/nm). (a and b) *S*_CD_ profile for DOPC in the absence of cholesterol. (c and d) *S*_CD_ profile for DOPC in the cholesterol-containing membranes (30 mol %). The horizontal axis indicates the carbon atom index (C2–C16). The assignment of the sn-1 and sn-2 hydrocarbon chains is shown in Figure S1a. Error bars represent standard deviations.

To further investigate electric-field-induced structural rearrangements, we analyzed the mass density profiles along the membrane normal (*z*-axis) for selected atomic groups. As shown in Figure 3a, in pure DOPC membranes, the application of ***E***_Horz_ led to subtle but reproducible increases in the peak density of headgroup atoms (P, N, and glycerol-linked carbons), suggesting tighter lipid packing. In contrast, ***E***_Vert_ had a negligible influence on the spatial distribution of these atoms. The density of hydrocarbon tail atoms (C and H in sn-1 and sn-2 chains) remained largely unaffected under either field, indicating minimal vertical displacement of the lipid tails in cholesterol-free membranes. Individual profiles for each atom are listed in Figure S5. In cholesterol-containing membranes (30 mol %, Figure 3b,c), ***E***_Horz_ induced a redistribution of cholesterol molecules. Specifically, a reduction in cholesterol density was observed near the bilayer midplane (*z* = 0 nm) coupled with a slight increase in density near the headgroup region (Figure 3c). These observations suggest that ***E***_Horz_ causes cholesterol to reorient or shift away from the bilayer center, potentially increasing the ordering of the surrounding lipids. Notably, ***E***_Horz_ predominantly acted on cholesterol molecules with minimal structural changes on the DOPC tail. Overall, these density analyses reinforce the conclusion that ***E***_Horz_ induces lateral compression and enhanced lipid tail ordering, whereas ***E***_Vert_ shows a limited impact on membrane organization at the molecular level.

**Figure 3:**
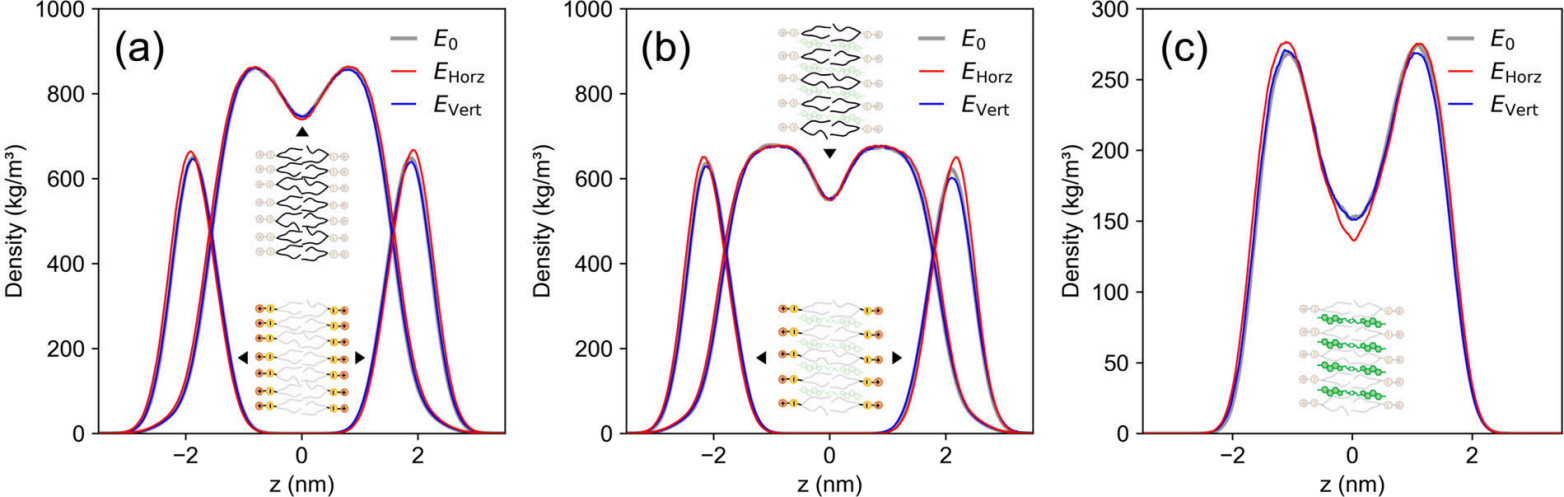
Mass density profiles along the membrane normal (*z*-axis) under different electric field conditions. Gray: no field; red: ***E***_Horz_ = 0.05 V/nm; blue: ***E***_Vert_ = 0.05 V/nm. (a) DOPC-only bilayer: overlaid density profiles of headgroup atoms (C, P, N, O) and hydrophobic tail atoms (C, H). (b) DOPC component in cholesterol-containing bilayer (30 mol %). (c) The cholesterol component in the 30 mol % bilayer. All profiles are centered at *z* = 0 nm based on the average position of phosphate atoms.

To examine the field-strength dependence of these responses, we performed additional simulations at doubled field intensity (0.10 V/nm) for the 30 mol % cholesterol bilayer (Figures S6–S8). The trends observed at 0.05 V/nm were amplified at 0.10 V/nm. ***E***_Horz_ and combined fields (***E***_Horz_ + ***E***_Vert_) induced a more pronounced reduction in APL, while ***E***_Vert_ produced only a minimal effect (Figure S6). Acyl chain order parameters further increased under ***E***_Horz_ and combined field conditions, whereas ***E***_Vert_ again produced negligible changes (Figure S7). Density profiles revealed a clear outward shift of the DOPC headgroup and glycerol atoms, accompanied by a distinct reduction in cholesterol density near the bilayer midplane (*z* = 0 nm) (Figure S8). Notably, this central density reduction is concentrated in cholesterol, with minimal displacement of DOPC tails. These additional simulations confirm that lipid bilayers exhibit structural responses dependent on both field orientation and intensity, reinforcing the conclusions drawn from our primary 0.05 V/nm simulations.

To experimentally evaluate structural changes in lipid bilayers under applied electric fields, PLBs were formed across a microaperture in a Teflon film and imaged using bright-field microscopy (Figure 4a, see SI and Scheme S1 for details). The edge of the aperture was coated with an organic solvent, which remained as a bulk organic phase (torus) surrounding the PLBs (Figure 4b,c). The membrane area remained nearly constant throughout the vertical voltage sweep. As shown in Figure 4b, no significant differences were observed between 0 mV and any of the vertical membrane voltages (*V*_Vert_) from −200 to 200 mV, indicating that ***E***_Vert_ did not measurably alter the membrane area (see Table S2). This observation is consistent with MD simulations, which showed no significant changes in the membrane area, bilayer thickness, or acyl chain order under ***E***_Vert_. Taken together, these findings indicate that ***E***_Vert_ at the relatively low field strengths investigated here, 0.05 V/nm in simulations and up to approximately ±0.04 V/nm in experiments (corresponding to a *V*_Vert_ of ±0.2 V across the membrane thickness of ∼5 nm), exerts a minimal mechanical influence on lipid bilayers. It is worth noting that at sufficiently high ***E***_Vert_ (e.g., >0.5 V/nm), electroporation and other effects are known to occur, highlighting that ***E***_Vert_ can affect membrane properties in different regimes.^32–34^

**Figure 4:**
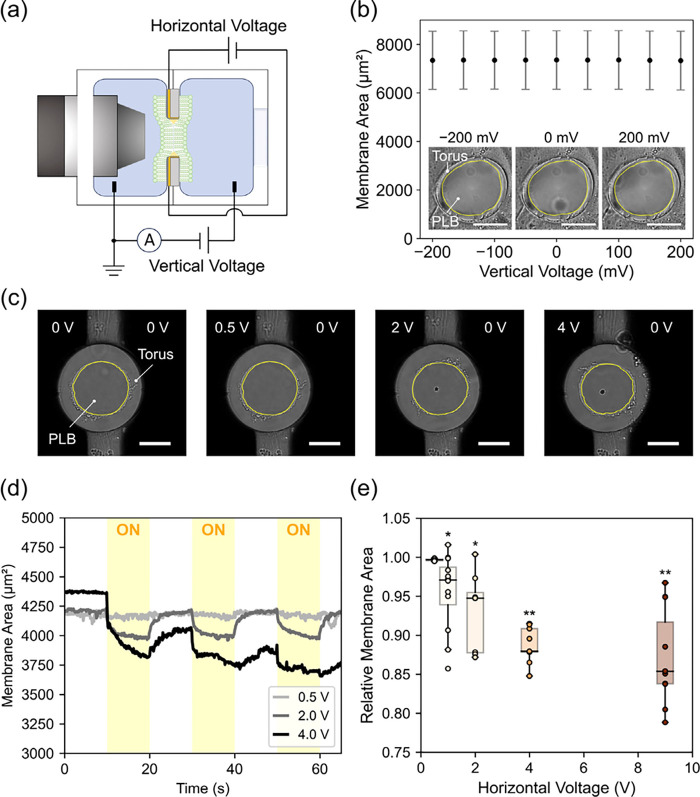
Experimental evaluation of membrane area changes in PLBs under *V*_Vert_ and *V*_Horz_ application. (a) Schematic top view of the experimental setup (not to scale). (b) Membrane area observations under different *V*_Vert_ conditions, with corresponding bright-field images. Each data point represents the mean ± SEM (*n* = 9 membranes) of the area averaged over the last 0.5 s of each 1-s *V*_Vert_ application. No statistically significant differences were observed between any voltage condition and the reference (*V*_Vert_ = 0 mV), as determined by a paired *t* test (see Table S2). Bright-field images show representative PLBs during application of *V*_Vert_ at −200 mV, 0 mV, and +200 mV (effective ***E***_Vert_ = – 0.04 V/nm, 0 V/nm, +0.04 V/nm; calculated by dividing *V*_Vert_ by ∼5 nm membrane thickness). The yellow line indicates the boundary between the lipid bilayer and the solvent torus. Scale bar: 50 μm. (c) Representative bright-field images during application of *V*_Horz_ at 0.5, 2, and 4 V (effective ***E***_Horz_ = 5 × 10^–6^ V/nm, 2 × 10^–5^ V/nm, 4 × 10^–5^ V/nm; calculated by dividing *V*_Horz_ by 100 μm aperture diameter). Scale bar: 50 μm. (d) Time course of membrane area under cyclic *V*_Horz_ application (10 s ON, 10 s OFF). (e) Relative membrane area vs *V*_Horz_ plot. Applied *V*_Horz_ = 0, 0.5, 2, 4, and 9 V (effective ***E***_Horz_ = 0 V/nm, 5 × 10^–6^ V/nm, 2 × 10^–5^ V/nm, 4 × 10^–5^ V/nm, and 9 × 10^–5^ V/nm). Area ratios were calculated by averaging the membrane area during 8–9 s of each ON phase and dividing it by the average area during the initial OFF phase (first 8–9 s segment). Each box indicates the interquartile range; horizontal lines indicate the median; individual data points are overlaid. Statistical significance was assessed using the Wilcoxon signed-rank test, comparing the ON and OFF area values for each condition (see Tables S3 and S4). Asterisks denote significance (*p* < 0.05: *, *p* < 0.01: **). Error bars indicate one standard deviation.

We next examined the effect of *V*_Horz_ on the membrane area. Two electrodes for applying *V*_Horz_ were deposited on the contralateral edges of the aperture in a Teflon film (Figure 4c). A *V*_Horz_ of DC 0.5–9 V was applied to the PLBs in a cyclic ON/OFF sequence, while the *V*_Vert_ was maintained at 0 mV. This corresponds to an estimated ***E***_Horz_ of 9 × 10^–^ ^5^ V/nm at 9 V, calculated by dividing the applied *V*_Horz_ by the ∼100 μm aperture diameter. As shown in Figure 4d, moderate *V*_Horz_ (0.5–2 V) induced reversible membrane contraction during the ON phase, followed by near-complete recovery during the OFF phase. At higher voltages (4 and 9 V), the *V*_Horz_-induced reduction in membrane area became too pronounced to recover within 10-s OFF intervals and exhibited a hysteresis effect during subsequent ON cycles^51–54^ (see Movie S1). Figure 4e summarizes the effect of *V*_Horz_ on the membrane area. Relative membrane area was calculated as the ratio of the average membrane area during each ON period (8–9 s) to the average membrane area during the first OFF period (8–9 s) before *V*_Horz_ was applied. The membrane area decreased with an increase in the *V*_Horz_. The median of the relative membrane area was 0.997 at 0.5 V, 0.971 at 1 V, 0.948 at 2 V, 0.879 at 4 V, and 0.854 at 9 V. Statistically significant reductions in membrane area were observed at *V*_Horz_ ≥ 1 V (*p* < 0.05, see Table S3 and S4). These results demonstrate that *V*_Horz_ consistently induces lateral compressive stress in the membrane, in agreement with MD simulations showing ***E***_Horz_-induced membrane contraction and enhanced lipid tail ordering.

To explore the potential influence of *V*_Vert_ on this behavior, we performed additional experiments in which *V*_Horz_ was cyclically applied under *V*_Vert_ = 100 mV (Figure S9). The membrane displayed area reduction and recovery similar to those of the *V*_Vert_ = 0 mV case. Notably, under *V*_Vert_ = 100 mV, the membrane area returned to its initial value within 10-s OFF intervals even during 4 V *V*_Horz_ cycles, whereas in the absence of *V*_Vert_, recovery was incomplete. This suggests that *V*_Vert_ may facilitate membrane relaxation following *V*_Horz_ application.

Our previous study showed that *V*_Horz_ promotes the opening of the Na_V_1.5 channel.^30,31^ Although this channel is primarily gated by the conventional transmembrane potential (*V*_Vert_), making a direct electrical effect plausible, the MD results and prior reports of its tension sensitivity^55^ indicate an additional mechanism. By modification of the bilayer structure, tension, and bending rigidity, ***E***_Horz_ may indirectly modulate channel gating. Such a mechanism could extend to a wide range of membrane proteins, potentially influencing diverse cellular processes.

In summary, we investigated the effect of ***E***_Horz_ on the structure of DOPC–cholesterol bilayers using all-atom MD simulations and bright-field imaging of PLBs with identical composition. Simulations revealed that ***E***_Horz_ compresses the membrane area and enhances lipid tail alignment, as indicated by reductions in APL and increases in order parameters, across cholesterol concentrations up to 30 mol %. In contrast, ***E***_Vert_ produced negligible effects on the membrane area and acyl chain order under the same field strength (0.05 V/nm). Experimental observations further supported the simulation findings: voltage-dependent membrane contraction was induced by *V*_Horz_ but not by *V*_Vert_, confirming the orientation-specific electromechanical response of the lipid bilayers. The close qualitative agreement between simulation and experiment underscores the pronounced electromechanical anisotropy of lipid membranes and highlights ***E***_Horz_ as a physiologically relevant but still underexplored force.

Notably, the effective ***E***_Horz_ in our experiments was estimated to be ∼9 × 10^–^ ^5^ V/nm, whereas the corresponding field in MD simulations was 0.05 V/nm, approximately 500× larger. Despite this magnitude difference, the experimental lipid bilayer exhibited a larger decrease in the membrane area upon application of *V*_Horz_. This difference may be attributed to the PLB setup, which employs *n*-hexadecane as a solvent to stabilize the bilayer. A fraction of this hydrophobic solvent is known to remain in the bilayer, altering its mechanical properties^50,56^ and plausibly amplifying ***E***_Horz_-induced contraction. Nevertheless, the same qualitative response was observed in solvent-free MD simulations, indicating that the ***E***_Horz_ effect reflects intrinsic lipid mechanics rather than an experimental artifact.^57^ It should also be noted that the nonpolarizable force field used in our MD simulations may underestimate membrane susceptibility to weak fields due to the absence of polarizability and local polarization.^56^ Future work employing polarizable force fields might enable a more quantitative comparison and provide predictive insight into three-dimensional, field-induced membrane structural changes under physiologically relevant conditions.

Finally, our simulations also revealed that ***E***_Horz_ drives cholesterol redistribution away from the bilayer center (*z* = 0 nm), reducing the central density of cholesterol and potentially altering local lipid packing and membrane rigidity.^58^ Such reorganization could alter hydrophobic coupling between the bilayer and embedded proteins, thereby influencing protein conformational equilibria and function.^57,59^ It may also modulate the activity of proteins involved in cholesterol translocation across membranes,^60,61^ potentially affecting membrane proteins whose function depends on cholesterol binding, as exemplified by the β_2_-adrenergic receptor^62^ and general cholesterol-interacting proteins.^63^ Altogether, these findings suggest that ***E***_Horz_ can reshape the membrane architecture and function, with potential implications for processes ranging from epithelial organization to neuronal signaling.

## Supplementary Material





## Notes

Full simulation parameters and system composition; experimental setup and protocols for PLB experiments; image acquisition and contour-tracking algorithms for membrane area quantification; statistical analysis results (Wilcoxon signed-rank and *t* tests) applied to area changes under different voltage conditions; supplementary figures (Figures S1–S9), scheme (Scheme S1), and tables (Tables S1–S4) (PDF)
Movie S1: bright-field time-lapse of membrane area contraction upon application of *V*_Horz_ (4 V) with cyclic ON/OFF (10 s, shown at 4× speed) (MOV)

## Abbreviations

MD: Molecular Dynamics
PLB: Planar lipid bilayers
***E***_Horz_: Horizontal electric fields
***E***_Vert_: Vertical electric fields
*V*_Horz_: Horizontal membrane voltage
*V*_Vert_: Vertical membrane voltage
APL: Area per lipid
DOPC: 1,2-Dioleoyl-*sn*-glycero-3-phosphocholine
