# Supplementary material for: Distinct Structural Responses of Lipid Bilayers to Horizontal and Vertical Electric Fields
Source: J Phys Chem Lett. 2025 Oct 6;16(41):10647–54. doi: 10.1021/acs.jpclett.5c02764 (PMC12536441; doi:10.1021/acs.jpclett.5c02764)
Supplement: Supplementary file 1 [file jz5c02764_si_001.pdf]

Supporting Information for

Distinct Structural Responses of Lipid Bilayers to Horizontal and Vertical Electric Fields

Authors

Hironori Kageyama [1, 2], Maki Komiya [2], Eiji Yamamoto [3, *], Ayumi Hirano-Iwata [1, 2, 4, *]

Affiliations

[1] Graduate School of Biomedical Engineering, Tohoku University, 6-6-12, Aramaki Aza Aoba, Aoba-ku, Sendai, Miyagi 980-8579, Japan

[2] Research Institute of Electrical Communication (RIEC), Tohoku University, 2-1-1, Katahira, Aoba-ku, Sendai, Miyagi 980-8577, Japan

[3] Department of System Design Engineering, Keio University, 3-14-1 Hiyoshi, Kohoku-ku, Yokohama, Kanagawa 223-8522, Japan

[4] Advanced Institute for Materials Research (WPI-AIMR), Tohoku University, 2-1-1 Katahira, Aoba-ku, Sendai, Miyagi 980-8577 Japan

*** Contact the corresponding authors**

Ayumi Hirano-Iwata

Research Institute of Electrical Communication (RIEC), Tohoku University, 2-1-1, Katahira, Aoba-ku, Sendai, Miyagi 980-8577, Japan

Email: ayumi.hirano.a5@tohoku.ac.jp

Eiji Yamamoto

Department of System Design Engineering, Keio University, 3-14-1 Hiyoshi, Kohoku-ku, Yokohama, Kanagawa 223-8522, Japan

Email: eiji.yamamoto@sd.keio.ac.jp

Table of contents

- **MD Simulation Details**

  —Simulation Parameters

  —System Composition

- **Bright-field Imaging of Planar Lipid Bilayers (PLBs)**

  —Membrane Preparation

  —Experimental Procedures

- **Supplementary Figures**
- **References**

- **MD Simulation Details**

Simulation Parameters

Each bilayer was embedded in a cubic periodic box, with water and ions added above and below the membrane. The systems were initially energy-minimized using the steepest descent algorithm for up to 5,000 steps with a convergence tolerance of 1000.0 kJ/mol/nm. Equilibration was performed for 100 ps under *NPT* conditions at 300 K and 1 bar. Temperature was regulated using the V-rescale thermostat[1], with separate coupling groups for the membrane and water/ions. Pressure was maintained using a Berendsen barostat[2] with semi-isotropic coupling (independent control in the *xy*-plane and z-direction), a coupling constant of 1.0 ps, and a compressibility of 4.5×10^{-5} bar^{-1}[3]. Initial velocities were generated from a Maxwell distribution at 300 K.

Following equilibration, production runs were carried out for 700 ns per system, with coordinates saved every 10 ps. The system was considered equilibrated after 100 ns based on the time variation of the area per lipid (APL), and subsequent frames were used for analysis (see Figure S2). During production, temperature and pressure were maintained using the V-rescale thermostat and the Parrinello–Rahman barostat[3], respectively.

Nonbonded interactions were treated using the Verlet cutoff scheme. Van der Waals interactions were truncated at 1.2 nm using a force-switch modifier starting at 1.0 nm. Electrostatics were computed using the Particle Mesh Ewald (PME) method[4] with a 1.2 nm cutoff. Bond constraints involving hydrogen atoms were applied using the LINCS algorithm[5].

External electric fields of 0.05 V/nm were applied along either the x-direction or z-direction, corresponding to horizontal and vertical electric field conditions, respectively.

System Composition

Table S1. Initial Composition of Simulated Lipid Bilayer Systems with Varying Cholesterol Molar Fractions. The table lists the number of DOPC and cholesterol molecules in each system. The cholesterol molar fraction is defined as mol% Chol. = $N_{Chol.}$ / (N_{DOPC} + $N_{Chol.}$) $\times$100.

System (mol%Chol.)	*N*-DOPC	*N*-Chol.	*N*-(DOPC+Chol.)	$L_x \cdot L_y$
DOPC + 0% Chol.	524	0	524	18261.4
DOPC + 10% Chol.	504	56	560	18684.4
DOPC + 20% Chol.	464	116	580	18490.4
DOPC + 30% Chol.	420	180	600	18237.0

- **Bright-field Imaging of Planar Lipid Bilayers (PLBs)**

Membrane Preparation

PLBs were formed using the folding method[6] across a microaperture (100-150 μm) fabricated in a Teflon film (thickness: 12.5 μm). The lipid solution (DOPC:cholesterol = 4:1 by weight) was dissolved in *n*-hexane. The aqueous phase consisted of 0.15 M KCl and 10 mM HEPES (pH 7.4, adjusted with KOH). Prior to bilayer formation, the edge of the microaperture was precoated with either squalane (Figure S1c) or *n*-hexadecane (Figure S1d): squalane for vertical membrane voltage (V_{Vert}) experiments, and *n*-hexadecane for V_{Horz} experiments.

The PLB formation process is illustrated in Scheme S1. In step (i), approximately 1 mL of KCl buffer was added to the bottom of each compartment in the Teflon chamber to form shallow pools below the aperture. In step (ii), 200 μL of lipid solution (5 mg/mL) was added to both sides and left undisturbed for 10 minutes. In step (iii), the buffer level was gradually raised on both sides to form a PLB at the aperture. Final buffer volumes were adjusted to 4 mL on the objective side and 6 mL on the opposite side. All experiments were performed at a controlled room temperature of 25 ± 1 °C (298.15 ± 1 K). After bilayer formation, membrane resistance was measured using a patch-clamp amplifier (Axopatch 200B), and only membranes exhibiting a resistance ≥250 GΩ were used for analysis.

Experimental Procedures

Bright-field imaging was conducted using a water-immersion objective lens (63×, NA = 1.0, ZEISS) inserted horizontally through the chamber wall. White light was illuminated from the opposite side through a quartz window, whose surface had been silanized with a long-chain perfluorocarbon, (tridecafluoro-1,1,2,2-tetrahydrooctyl)dimethylchlorosilane (PFDS)[7]. Images were acquired at 10 fps using a CMOS camera (ORCA-Fusion C1440, Hamamatsu Photonics). Image analysis was performed using the Snakes algorithm[8] implemented in Fiji/ImageJ. Prior to contour fitting, each image underwent histogram equalization, smoothing, edge enhancement, and brightness adjustment to improve boundary detectability. The initial contour was manually placed just inside the membrane edge. All image frames comprising a

single movie were analyzed using the same initial contour, preprocessing steps, and number of optimization iterations to ensure consistency in area quantification. To avoid divergence of the contour due to image artifacts such as bubbles or blurred boundaries, the curvature of the evolving contour was restricted during optimization.

In the V_{Vert} experiments, squalane was chosen as a solvent for its low bilayer permeability, minimizing solvent extrusion artifacts. This setup allowed us to reliably monitor the membrane area while varying V_{Vert} from –200 mV to +200 mV in 50 mV increments every 1 second. For each voltage step, we analyzed the membrane area during the latter 0.5 seconds of the 1-second application period, and calculated sample-wise averages (n = 9). These values were statistically compared to the area observed at 0 mV using paired t-tests (see Table S2).

Table S2. Statistical Comparison of Membrane Areas under V_{Vert} Conditions Relative to 0 mV

V_{Vert} (mV)	Mean	SEM	n	ΔArea (mean)	p-value	Significance
-200	7340.48	1197.55	9	14.44	0.309	ns
-150	7353.75	1202.61	9	1.16	0.768	ns
-100	7347.39	1193.92	9	7.52	0.561	ns
-50	7351.07	1207.36	9	3.85	0.565	ns
0	7354.91	1204.58	9	-	-	-
50	7350.66	1209.02	9	4.25	0.516	ns
100	7356.80	1206.42	9	1.88	0.662	ns
150	7340.58	1206.51	9	14.33	0.143	ns
200	7330.48	1210.94	9	24.43	0.090	ns

In the V_{Horz} experiments, PLBs were formed across a microaperture in a Teflon film equipped with two electrodes. The electrode-equipped film was fabricated according to the procedure described in ref.[9], with the exception that a Ti/Au/Ti multilayer was used instead of a single Ti layer. *N*-hexadecane was used in place of squalane because preliminary trials with squalane resulted in frequent membrane rupture upon V_{Horz} application. This increased fragility is likely attributable to the higher bending rigidity and lower compressibility of squalane-based membranes compared to those formed in *n*-hexadecane. Squalane, being a highly viscous, long-chain hydrocarbon (Figure S1c), forms thinner and more tightly packed bilayers with reduced thickness fluctuations, which leads to a more rigid and less deformable membrane core[10,11]. As a result, squalane-based bilayers resist deformation but are more susceptible to rupture under in-plane compressive stress. In contrast, *n*-hexadecane, with its lower viscosity and higher fluidity with lipids, yields more robust membranes that can tolerate greater mechanical strain under V_{Horz}[12,13]. After the formation of lipid bilayers, a DC horizontal voltage was toggled ON and OFF every 10 seconds, while the vertical voltage was held constant at 0 mV. Since normality could not be assumed for the ON–OFF area differences (Shapiro–Wilk test, $p < 0.05$ at 2.0 V and 9.0 V, see Table S3), the non-parametric Wilcoxon signed-rank test was employed (Table S4).

Table S3. Shapiro-Wilk test for normality of membrane area changes under V_{Horz} applications.

V_{Horz} (V)	n	p-value	Normality
0.5	6	0.82341	Yes
1	12	0.21372	Yes
2	8	0.04416	No
4	9	0.49033	Yes
9	11	0.00001	No

Table S4. Wilcoxon signed-rank test of membrane area changes between ON and OFF phases under V_{Horz} application.

V_{Horz} (V)	n	ΔArea (mean)	p-value	Significance
0.5	6	883.318	0.09375	ns
1	12	13263.348	0.01611	*
2	8	27473.091	0.01562	*
4	9	24346.081	0.00391	**
9	11	14187.727	0.00098	**

- **Supplementary Figures**

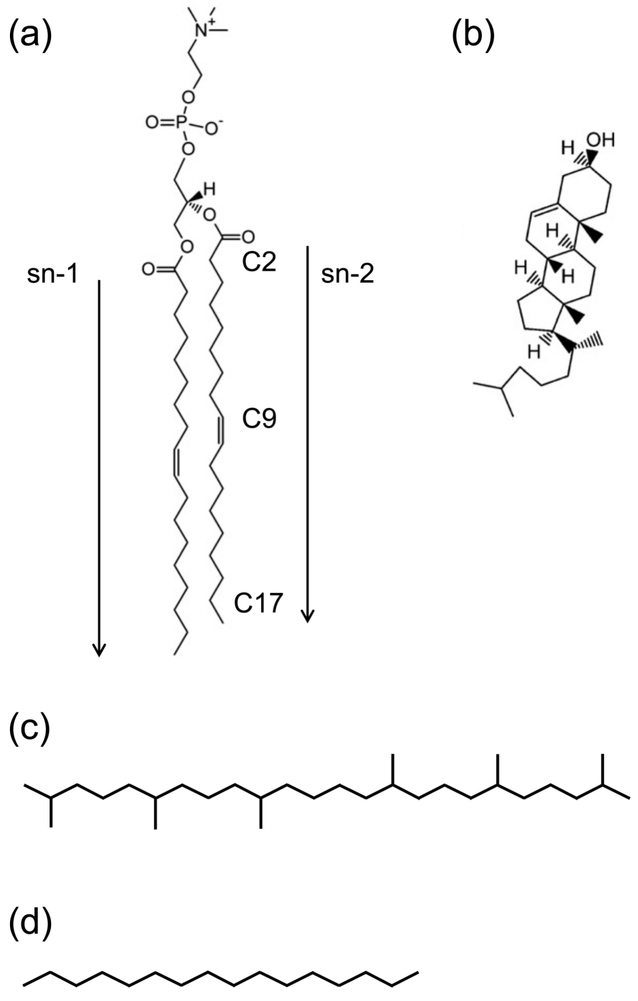

Figure S1. Figure S1. Molecular structures of the major components used in this study. (a) 1,2-dioleoyl-sn-glycero-3-phosphocholine (DOPC), (b) cholesterol, (c) squalane, and (d) *n*-hexadecane.

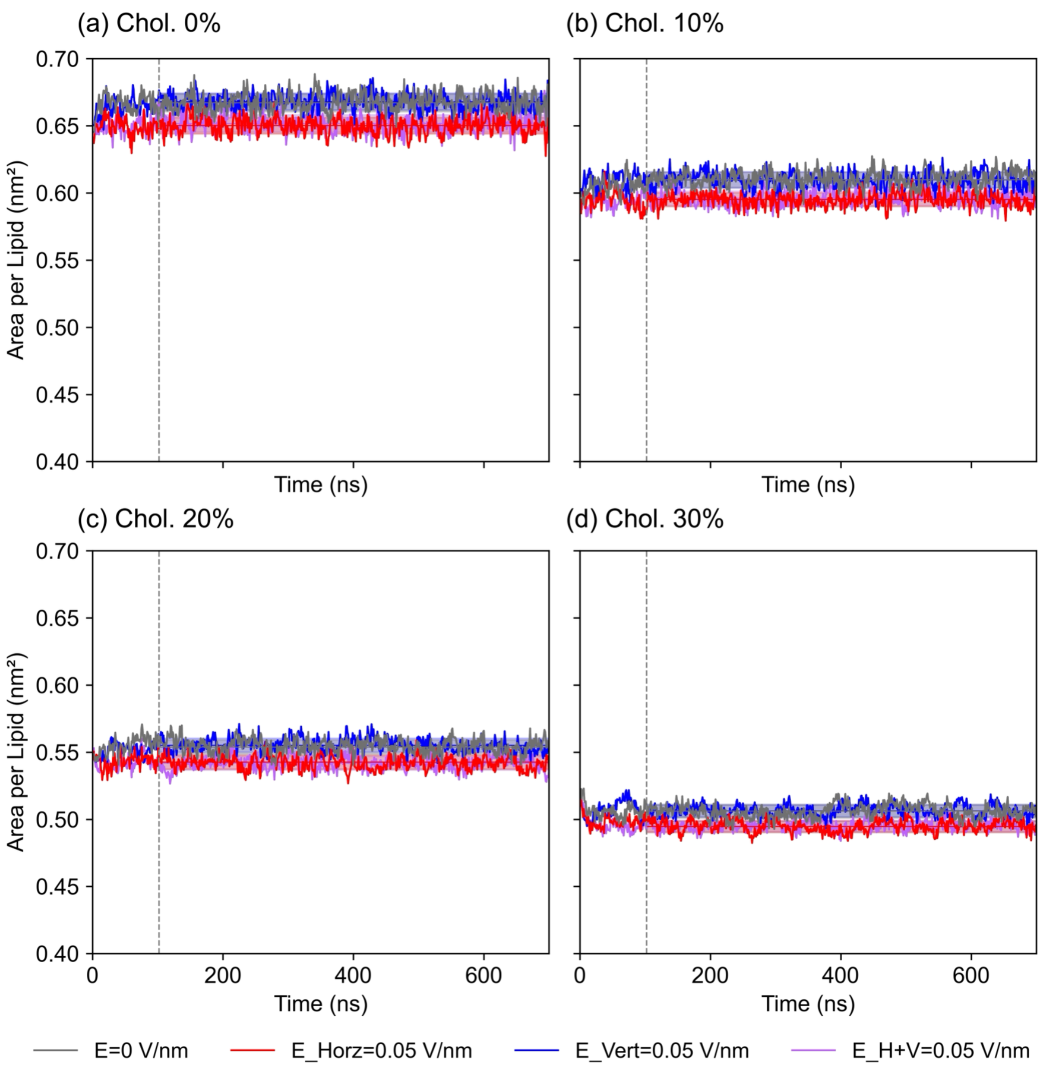

Figure S2. Time variation of the area per lipid (APL). Each subplot corresponds to a different cholesterol concentration (mol%). Red, blue, and purple lines represent the behavior under horizontal, vertical, and combined electric fields, respectively. The gray dashed line indicates the simulation time of 100 ns. The system was considered equilibrated after 100 ns based on the time variation of APL.

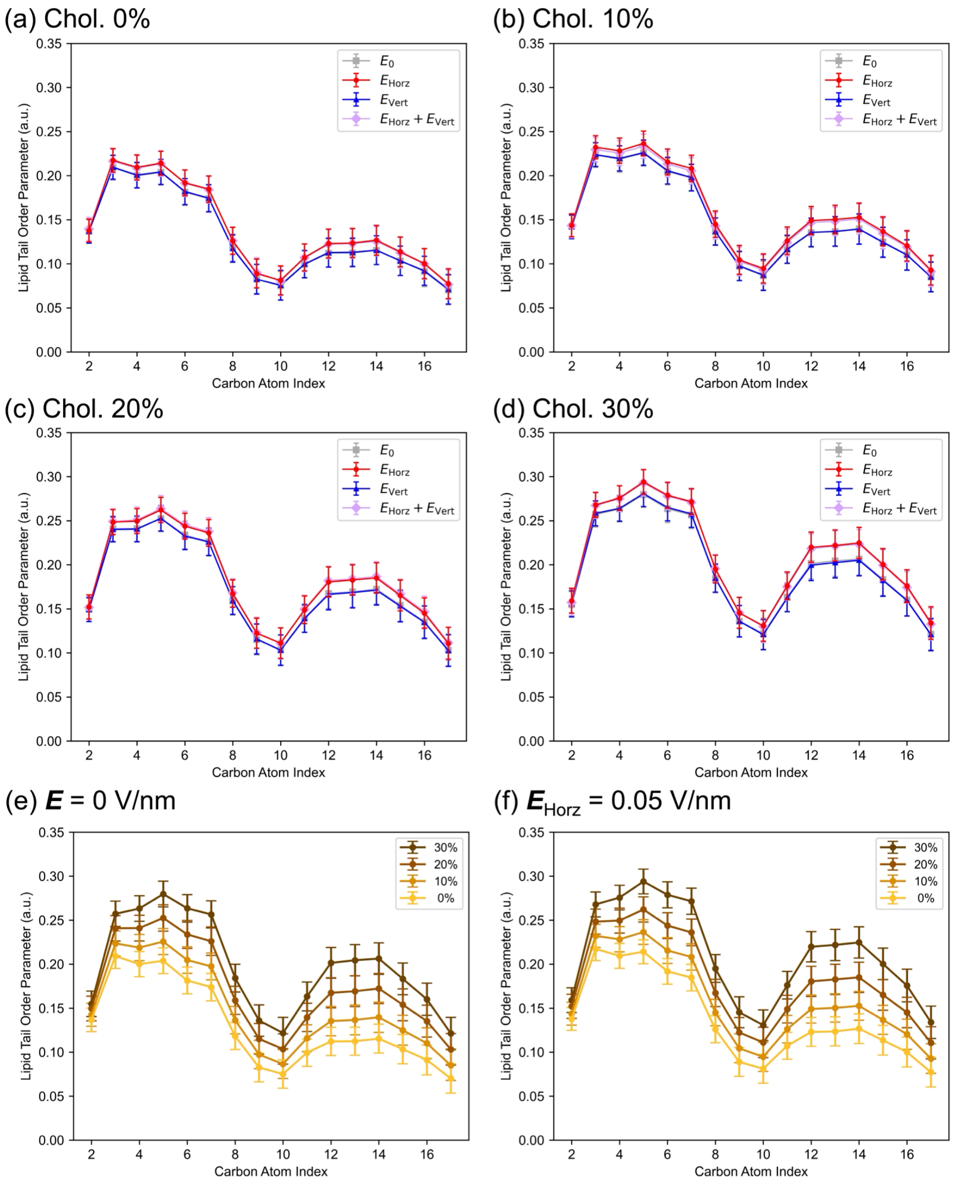

Figure S3. Segmental order parameters ($|-S_{\mathrm{CD}}|$) of DOPC sn-1 hydrocarbon chains under varying electric field and cholesterol conditions. (a–d) Order parameter profiles at different cholesterol concentrations (0, 10, 20, and 30 mol%). (e) Comparison of $|-S_{\mathrm{CD}}|$ profiles across cholesterol concentrations under $\boldsymbol{E}$ = 0 V/nm. (f) Comparison of $|-S_{\mathrm{CD}}|$ profiles across cholesterol concentrations under $\boldsymbol{E}_{\mathrm{Horz}}$ = 0.05 V/nm. Error bars represent standard deviation. The horizontal axis indicates carbon atom indices (C2–C16).

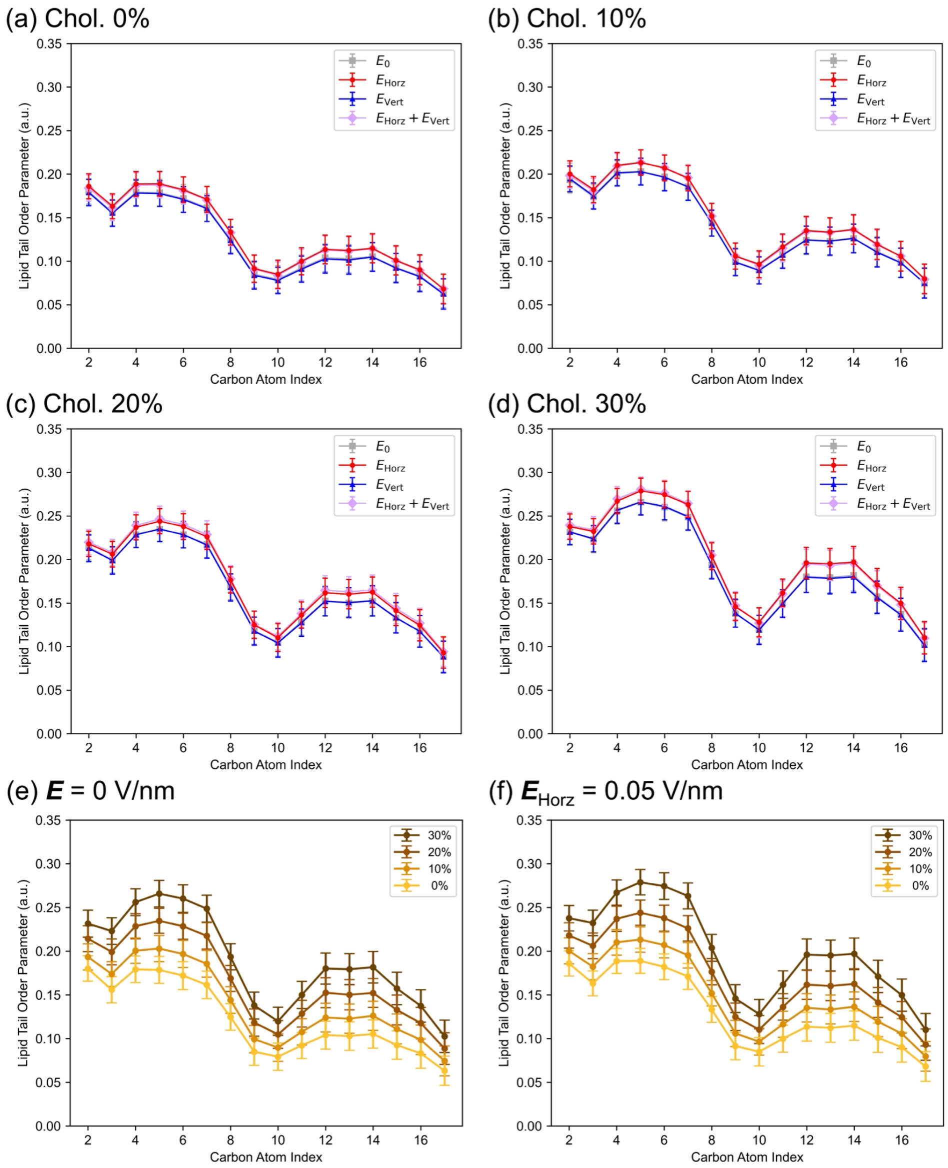

Figure S4. Segmental order parameters ($|-S_{CD}|$) of DOPC sn-2 hydrocarbon chains under varying electric field and cholesterol conditions. (a–d) Order parameter profiles at different cholesterol concentrations (0, 10, 20, and 30 mol%). (e) Comparison of $|-S_{CD}|$ profiles across cholesterol concentrations under $\boldsymbol{E}$ = 0 V/nm. (f) Comparison of $|-S_{CD}|$ profiles across cholesterol concentrations under $\boldsymbol{E}_{Horz}$ = 0.05 V/nm. Error bars represent standard deviation. The horizontal axis indicates carbon atom indices (C2–C16).

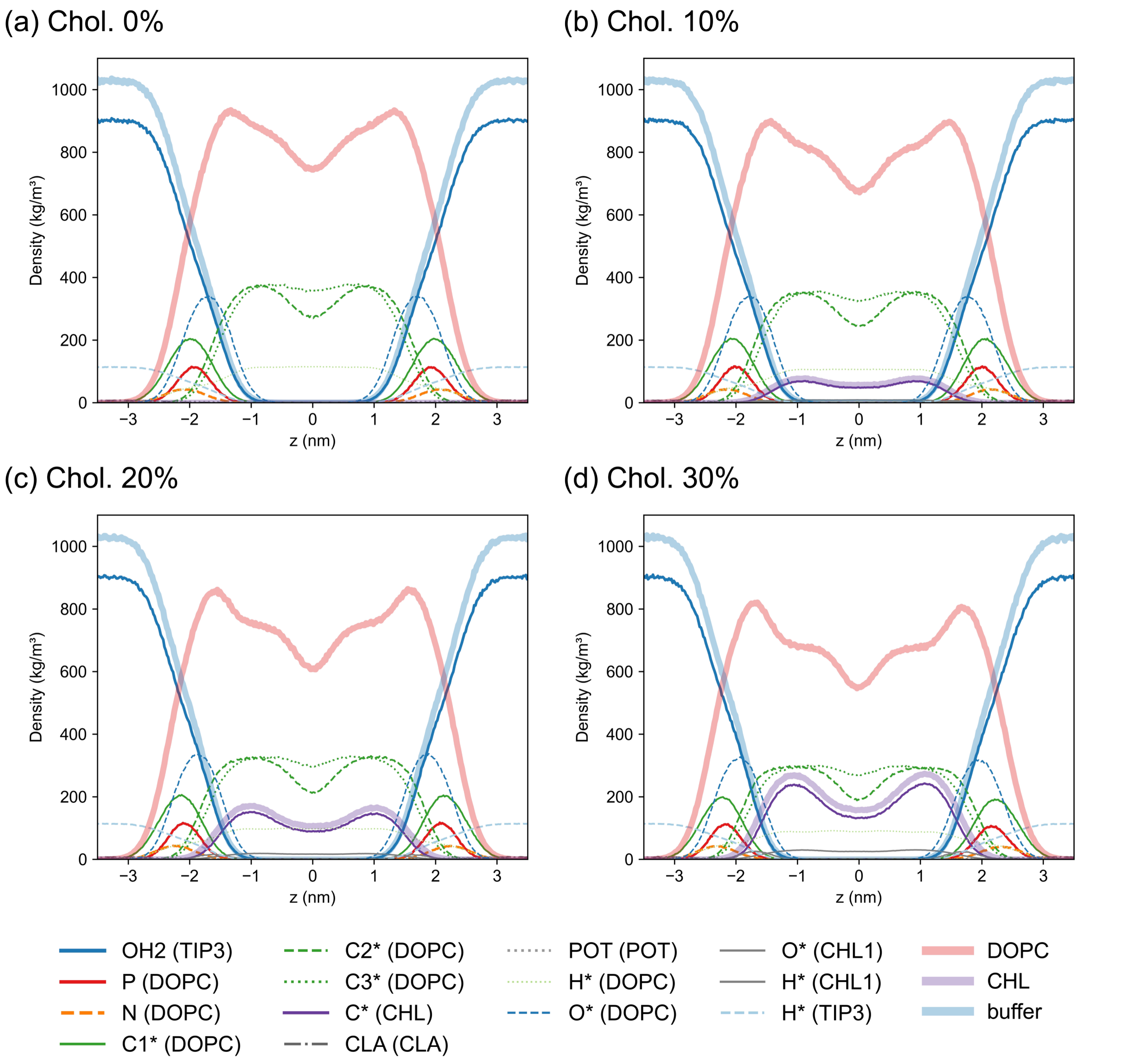

Figure S5. Mass Density profiles. (a) pure DOPC membrane, (b-d) Cholesterol-containing membrane (10-30%).

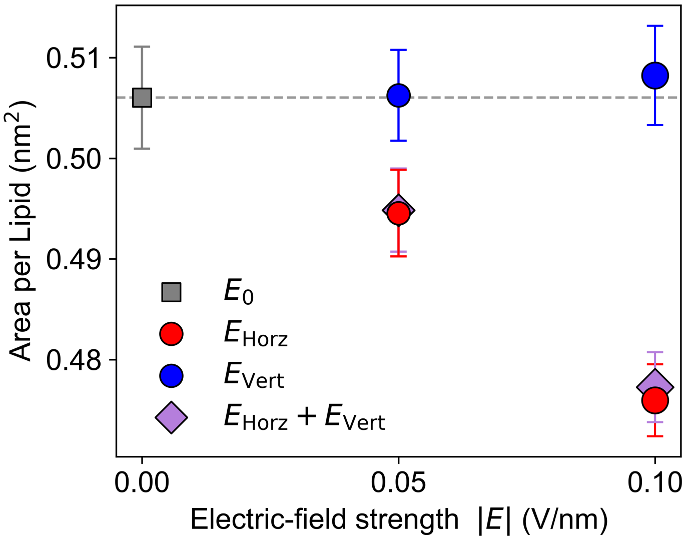

Figure S6. Area per lipid (APL) for a DOPC bilayer containing 30 mol% cholesterol as a function of the applied electric-field magnitude. Gray squares denote the field-free reference (***E***$_0$); red circles, blue circles, and purple rhombi correspond to fields applied along the x-axis (***E***$_{Horz}$), z-axis (***E***$_{Vert}$), and simultaneously along both axes (***E***$_{Horz}$ + ***E***$_{Vert}$), respectively. Marker size scales with field intensity. The horizontal dashed line represents the mean APL of the ***E***$_0$ trajectory, highlighting deviations induced by the external field. Symbols are plotted at the nominal field strengths of 0, 0.05, and 0.10 V/nm; error bars show the standard deviation of block-averaged APL values over the final 600 ns of each simulation.

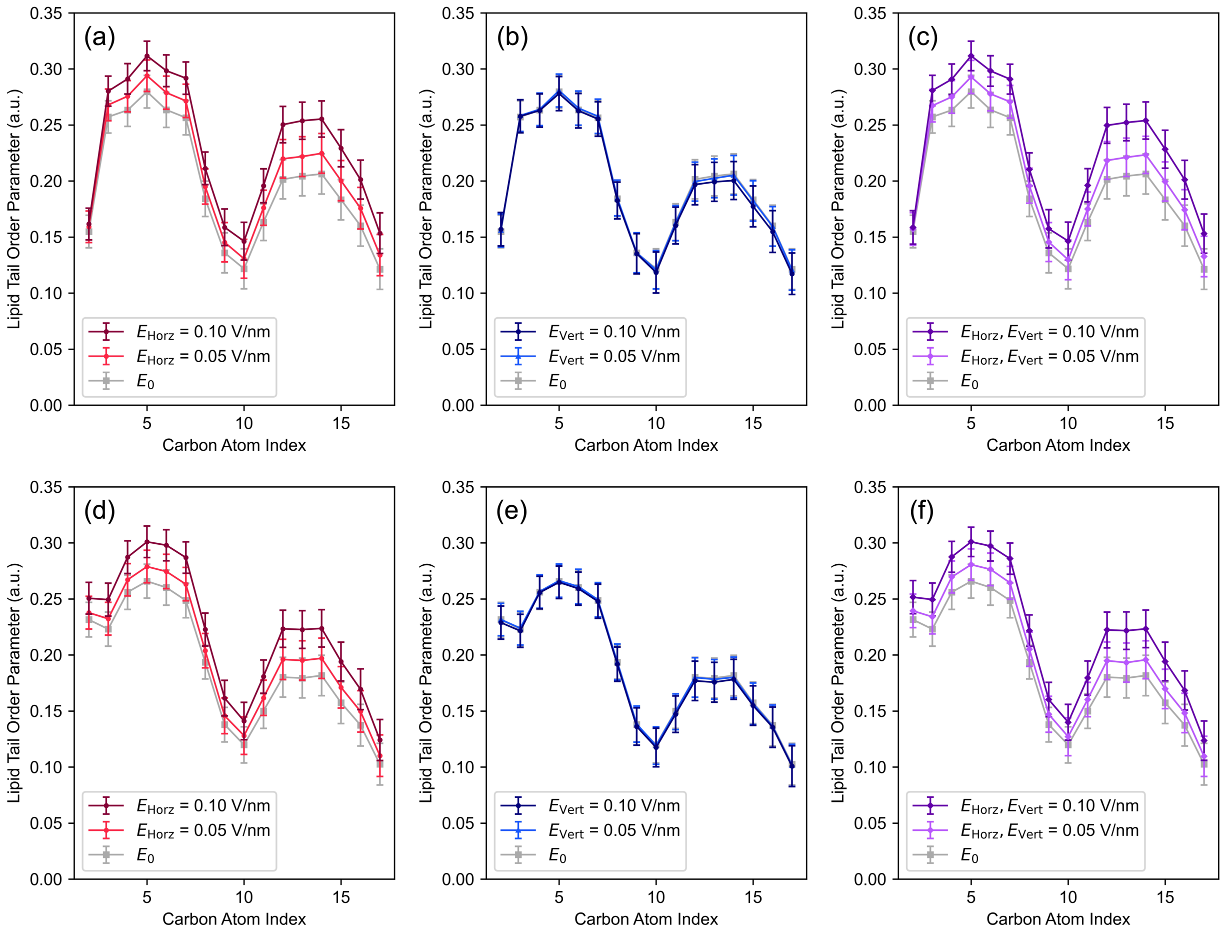

Figure S7. Segmental order parameters (S_{CD}) of DOPC hydrocarbon chains under an electric field of 0.10 V/nm for the 30 mol% cholesterol bilayer. (a–c) sn-1 hydrocarbon chains; (d-f) sn-2 hydrocarbon chains. Gray: no field; red: E_{Horz} = 0.05 V/nm; dark red: E_{Horz} = 0.10 V/nm; blue: E_{Vert} = 0.05 V/nm; dark blue: E_{Vert} = 0.10 V/nm; purple: E_{Horz} + E_{Vert} (each 0.05 V/nm); dark purple: E_{Horz} + E_{Vert} (each 0.10 V/nm). (a, d) E_{Horz} only; (b, e) E_{Vert} only; (c, f) combined E_{Horz} and E_{Vert}. Error bars represent standard deviation. The horizontal axis indicates carbon atom indices (C2–C16).

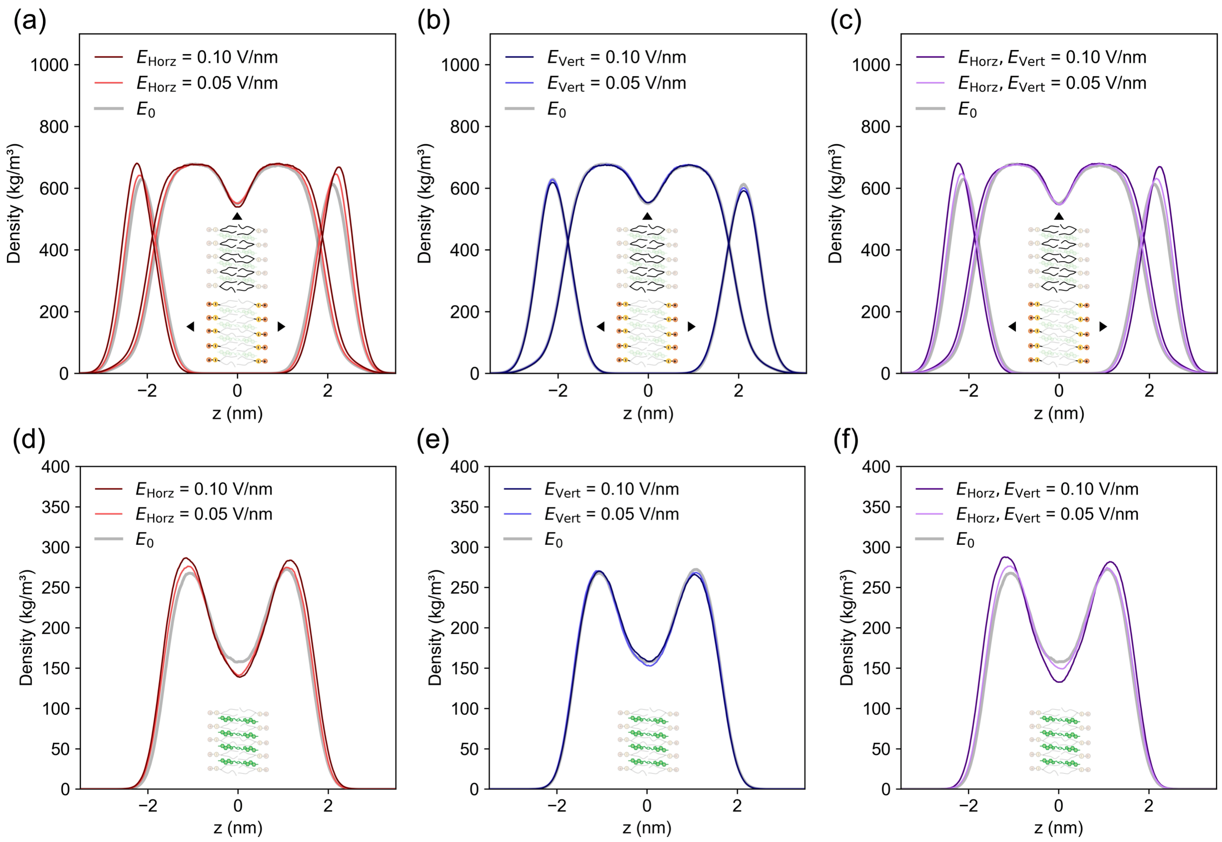

Figure S8. Mass density profiles at an electric field of 0.10 V/nm for the 30 mol% cholesterol bilayer. Profiles along the membrane normal (z-axis) for (a–c) DOPC and (d–f) cholesterol components. Gray: no field; red: $\boldsymbol{E}_{\mathrm{Horz}}$ = 0.05 V/nm; dark red: $\boldsymbol{E}_{\mathrm{Horz}}$ = 0.10 V/nm; blue: $\boldsymbol{E}_{\mathrm{Vert}}$ = 0.05 V/nm; dark blue: $\boldsymbol{E}_{\mathrm{Vert}}$ = 0.10 V/nm; purple: $\boldsymbol{E}_{\mathrm{Horz}}$ + $\boldsymbol{E}_{\mathrm{Vert}}$ (each 0.05 V/nm); dark purple: $\boldsymbol{E}_{\mathrm{Horz}}$ + $\boldsymbol{E}_{\mathrm{Vert}}$ (each 0.10 V/nm). (a, d) $\boldsymbol{E}_{\mathrm{Horz}}$ only; (b, e) $\boldsymbol{E}_{\mathrm{Vert}}$ only; (c, f) combined $\boldsymbol{E}_{\mathrm{Horz}}$ and $\boldsymbol{E}_{\mathrm{Vert}}$. All profiles are centered at z = 0 nm based on the average position of phosphate atoms.

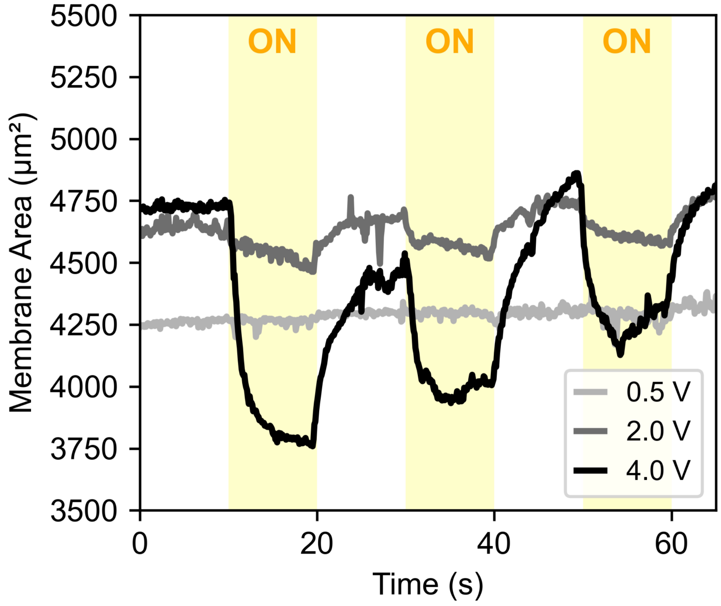

Figure S9. Experimental evaluation of membrane area changes in PLBs with combined V_{Vert} and V_{Horz} application. Time course of membrane area under V_{Vert} = 100 mV during cyclic V_{Horz} application (10 s ON, 10 s OFF). This experiment complements the data shown in Figure 4d (V_{Vert} = 0 mV), and the same PLB was used.

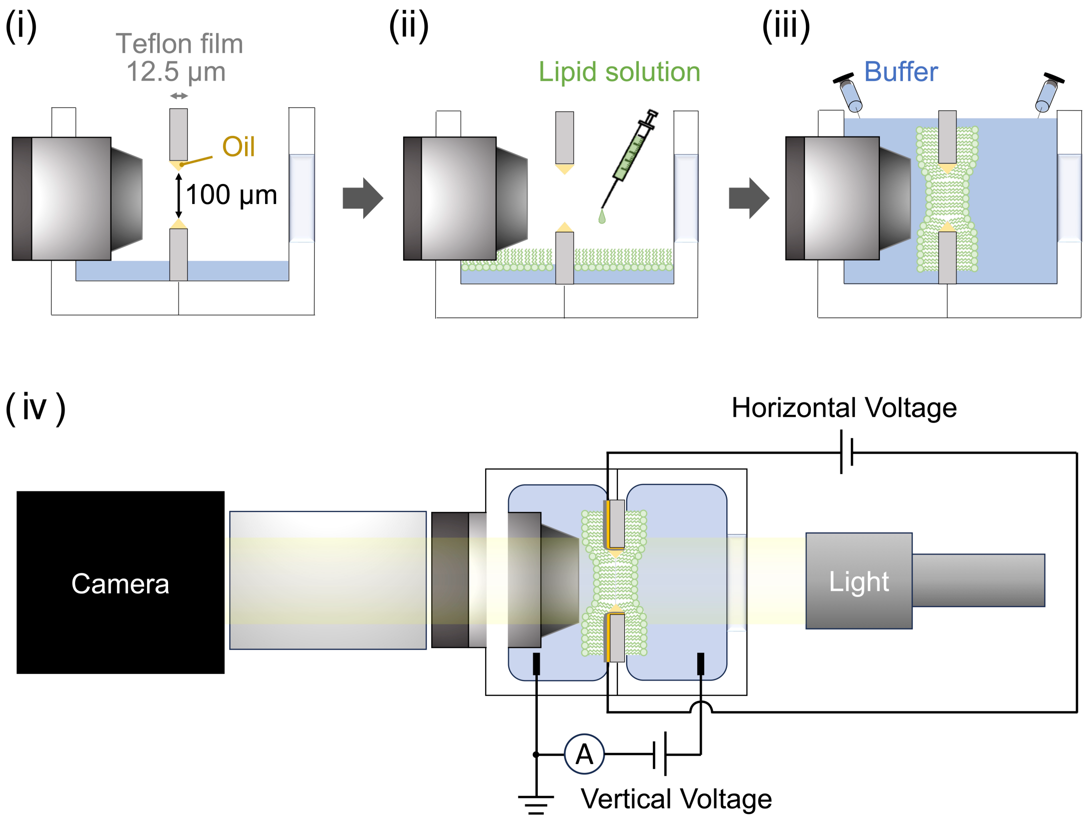

Scheme S1. Experimental setup for bright-field imaging of PLBs formed using the folding method (not to scale). Panels (i)–(iii) are side views illustrating the PLB formation process, and panel (iv) is a top view of the experimental setup. A lipid bilayer (thickness: ~5 nm) was formed across a microaperture (~150 µm) fabricated in a Teflon film (thickness: 12.5 µm), with an oil layer (light yellow) applied to bridge the thickness gap. The Teflon film, integrated with Ti/Au/Ti–SiO_2 electrodes for V_{Horz} application, was sandwiched between two halves of a custom-made Teflon chamber. Because panels (i)–(iii) depict a central cross-section, the electrodes located on either side (depth direction) are outside the illustrated plane. White light was illuminated through a quartz glass window opposite the objective lens.

REFERENCES

(1) Bussi, G.; Donadio, D.; Parrinello, M. Canonical Sampling through Velocity Rescaling. *The Journal of Chemical Physics* **2007**, *126* (1), 014101. https://doi.org/10.1063/1.2408420.

(2) Berendsen, H. J. C.; Postma, J. P. M.; Van Gunsteren, W. F.; DiNola, A.; Haak, J. R. Molecular Dynamics with Coupling to an External Bath. *The Journal of Chemical Physics* **1984**, *81* (8), 3684–3690. https://doi.org/10.1063/1.448118.

(3) Parrinello, M.; Rahman, A. Polymorphic Transitions in Single Crystals: A New Molecular Dynamics Method. *Journal of Applied Physics* **1981**, *52* (12), 7182–7190. https://doi.org/10.1063/1.328693.

(4) Essmann, U.; Perera, L.; Berkowitz, M. L.; Darden, T.; Lee, H.; Pedersen, L. G. A Smooth Particle Mesh Ewald Method. *The Journal of Chemical Physics* **1995**, *103* (19), 8577–8593. https://doi.org/10.1063/1.470117.

(5) Hess, B.; Bekker, H.; Berendsen, H. J. C.; Fraaije, J. G. E. M. LINCS: A Linear Constraint Solver for Molecular Simulations. *J. Comput. Chem.* **1997**, *18* (12), 1463–1472. https://doi.org/10.1002/(SICI)1096-987X(199709)18:12<1463::AID-JCC4>3.0.CO;2-H.

(6) MUELLER, P.; RUDIN, D. O.; TI TIEN, H.; WESCOTT, W. C. Reconstitution of Cell Membrane Structure in Vitro and Its Transformation into an Excitable System. *Nature* **1962**, *194* (4832), 979–980. https://doi.org/10.1038/194979a0.

(7) Yamaura, D.; Tadaki, D.; Araki, S.; Yoshida, M.; Arata, K.; Ohori, T.; Ishibashi, K.; Kato, M.; Ma, T.; Miyata, R.; Yamamoto, H.; Tero, R.; Sakuraba, M.; Ogino, T.; Niwano, M.; Hirano-Iwata, A. Amphiphobic Septa Enhance the Mechanical Stability of Free-Standing Bilayer Lipid Membranes. *Langmuir* **2018**, *34* (19), 5615–5622. https://doi.org/10.1021/acs.langmuir.8b00747.

(8) Kass, M.; Witkin, A.; Terzopoulos, D. Snakes: Active Contour Models. *Int J Comput Vision* **1988**, *1* (4), 321–331. https://doi.org/10.1007/BF00133570.

(9) Ma, T.; Sato, M.; Komiya, M.; Kanomata, K.; Watanabe, T.; Feng, X.; Miyata, R.; Tadaki, D.; Hirose, F.; Tozawa, Y.; Hirano-Iwata, A. Lateral Voltage as a New Input for Artificial Lipid Bilayer Systems. *Faraday Discuss.* **2022**, *233*, 244–256. https://doi.org/10.1039/D1FD00045D.

(10) Hladky, S. B.; Gruen, D. W. Thickness Fluctuations in Black Lipid Membranes. *Biophysical Journal* **1982**, *38* (3), 251–258. https://doi.org/10.1016/S0006-3495(82)84556-6.

(11) Beltramo, P. J.; Scheidegger, L.; Vermant, J. Toward Realistic Large-Area Cell Membrane Mimics: Excluding Oil, Controlling Composition, and Including Ion Channels. *Langmuir* **2018**, *34* (20), 5880–5888. https://doi.org/10.1021/acs.langmuir.8b00837.

(12) Venable, R. M.; Zhang, Y.; Hardy, B. J.; Pastor, R. W. Molecular Dynamics Simulations of a Lipid Bilayer and of Hexadecane: An Investigation of Membrane Fluidity. *Science* **1993**, *262* (5131), 223–226. https://doi.org/10.1126/science.8211140.

(13) Santinho, A.; Chorlay, A.; Foret, L.; Thiam, A. R. Fat Inclusions Strongly Alter Membrane Mechanics. *Biophysical Journal* **2021**, *120* (4), 607–617. https://doi.org/10.1016/j.bpj.2021.01.009.
